# The direct role of 5-lipoxygenase on tau pathology, synaptic integrity and cognition in a mouse model of tauopathy

**DOI:** 10.1038/s41398-017-0017-2

**Published:** 2017-12-18

**Authors:** Alana N. Vagnozzi, Phillip F. Giannopoulos, Domenico Praticò

**Affiliations:** Center for Translational Medicine, Department of Pharmacology, Lewis Katz School of Medicine, Temple University, Philadelphia, PA 19140 USA

## Abstract

Neurodegenerative tauopathies are characterized by pathological accumulation of highly phosphorylated isoforms of tau protein, which leads to progressive neuronal loss. Neuroinflammation often accompanies tau-driven diseases; however, the direct role of neuroinflammation in tauopathies remains unknown. The 5-lipoxygenase (5LO) is a pro-inflammatory enzyme, which produces several bioactive metabolites and is widely expressed in the central nervous system. Previously, our group showed that 5LO influences the Alzheimer’s disease (AD) phenotype of APP transgenic mice as well as a mouse model with plaques and tangles. However, whether this protein directly modulates tau phosphorylation and subsequent neuropathology remains to be fully investigated. In the current study, we provide evidence for an age-dependent and region-specific upregulation of the 5LO pathway (protein, message and activity) in a transgenic mouse model of tauopathy, the P301S line. In addition, we demonstrate that genetic deletion of 5LO in this mouse model results in significant memory improvement, reduces tau phosphorylation at specific epitopes as well as neuroinflammation and rescues synaptic pathology. In vitro studies confirmed that 5LO directly modulates tau phosphorylation at the same epitopes as for the brain tissues. Taken together, our data reveal an active involvement of the 5LO pathway in the development of the tauopathy phenotype and provide strong support to the hypothesis that this enzymatic protein should be considered a novel and viable therapeutic target for the treatment of human tauopathy.

## Introduction

The classic pathologic hallmark lesions in the Alzheimer’s disease (AD) brain are composed of pathogenic amyloid-beta (Aβ) peptides and tau protein, which lead to formation and deposits of Aβ plaques and neurofibrillary tangles, respectively^1^. In recent years, because of some disappointing results of the Aβ-centered therapies, research has focused on the tau protein and  its role in the pathogenesis of AD and related tauopathies. Human neurodegenerative tauopathies, which include a variety of diseases such as progressive supranuclear palsy (PSP), Pick’s disease and corticobasal degeneration, display progressive accumulation of hyper-phosphorylated tau protein and pathology together with cognitive impairments and synaptic loss in the absence of Aβ accumulation^2,3^.

While compelling evidence has emerged linking inflammatory pathways to neurodegeneration in both human and mouse models of tauopathy, the source of the neuroinflammation and whether it is a primary or secondary event in disease progression, has yet to be fully elucidated^4–6^.

The 5-lipoxygenase (5LO) is a pro-inflammatory enzyme that oxidizes free and esterified fatty acids to produce potent bioactive lipids, most of which are grouped under the name of leukotrienes (LTs)^7^. The 5LO is widely expressed in the central nervous system, where it localizes to both neuronal and glial cells. Previously, our group has demonstrated a role for 5LO in the development of the AD-like phenotype in transgenic mouse models characterized by a progressive accumulation of Aβ peptides, formation of neuritic plaques and behavioral impairments^8–10^. Interestingly, we also showed that modulation of 5LO expression levels influences the phenotype of a transgenic mouse model that develops Aβ plaques and tau tangles^11^. The purpose of this paper was to investigate the functional role that 5LO may have in directly modulating the development of the tau phenotype in a relevant mouse model of human tauopathy, the P301S mice. First, we assessed levels of 5LO in this model and showed that compared to wild-type (WT), transgenic mice display upregulation of the 5LO pathway (protein, message and activity) in an age-dependent and region-specific manner. Next, we generated P301S mice genetically deficient for 5LO, the P301S/5LO KO mouse line, and compared them to the regular tau mice by assessing learning and memory, tau phosphorylation and pathology, neuroinflammation and synaptic integrity. At the end of these studies, we found that the genetic absence of 5LO rescued behavioral deficits, tau phosphorylation, as well as synaptic pathology and neuroinflammation in P301S mice.

Taken together, our findings demonstrate a direct role of the 5LO pathway in modulating tau phosphorylation and neuropathology in a relevant mouse model of human tauopathy. They support the novel concept that the upregulation of 5LO in the central nervous system of this transgenic mouse model not only is an early event in pathogenesis but an important player with an active role in the development of the entire pathological phenotype.

## Materials and methods

### Animals

All animal procedures were approved by the Animal Care and Usage Committee, in accordance with the U.S. National Institutes of Health guidelines. The P301S mice (PS19 line) express human mutant microtubule-associated protein tau, *MAPT*, driven by the mouse prion protein (Prnp) promoter^4^. To produce our P301S/5LO KO mouse line, P301S mice were backcrossed ten times with mice lacking 5LO (5LO KO) on the same genetic background. The WT are age-matched P301S −/− controls. All animals were housed on a 12-h light/dark cycle in a pathogen-free environment and given regular chow and water ad libitum. Upon euthanasia, mice were perfused with ice-cold 0.9% phosphate-buffered saline (PBS) containing EDTA (2 mmol/l), pH 7.4. Brains were removed, gently rinsed in cold 0.9% PBS and immediately dissected into two halves. One half was stored at −80 °C for biochemistry, while the other half was fixed in 4% paraformaldehyde diluted in PBS, pH 7.4, for immunohistochemical studies.

### Behavioral tests

All the animals were handled for at least 3–4 consecutive days before testing. They were tested in random order and the experimenter conducting the tests was unaware of the genotype/group.

### Y-maze

The Y-maze apparatus consisted of three arms 32 cm (long) and 610 cm (wide) with 26-cm walls (San Diego Instruments, San Diego, CA). Testing was always performed in the same room and at the same time to ensure environmental consistency as previously described^12,13^. Briefly, each mouse was placed in the center of the Y-maze and allowed to explore freely during a 5-min session as a measure of spontaneous alternating behavior. The sequence and total number of arms entered were video-recorded. An entry into an arm was considered valid if all four paws entered the arm. An alternation was defined as three consecutive entries into three different arms (1, 2, 3, or 2, 3, 1, etc.). Percentage of alternation was calculated using the following formula: total alternation number/total number of entries−2) × 100.

### Novel object recognition

Mice were habituated to the open testing arena for three consecutive days (10 min each time). During the memory acquisition trial, each mouse was allowed to explore two identical objects for 10 min. For the memory retention phase, animals were exposed for 10 min to the presence of one similar object and one novel object (a different shape and color). Object exploration time was recorded when the mouse touched the object directly with its nose, mouth or forepaws. The discrimination index was calculated as the time spent near the new object divided by the cumulative time spent with both objects. Results are expressed as exploration percentage for each object.

### Morris water maze

To perform the Morris water maze (MWM), we used a white circular plastic tank (122 cm in diameter, walls 76 cm high), filled with water maintained at 22° ± 2 °C and made opaque by the addition of a nontoxic white paint, as previously described^12,13^. Mice were trained on four consecutive days to find a Plexiglas platform submerged in water from four different starting points. If they failed to find the platform within 60 s, they were manually guided to the platform and allowed to remain there for 15 s. Mice were trained to reach a training criterion of 20 s (escape latency). Mice were assessed in the probe trial, which consisted of a free swim lasting for 60 s without the platform, 24 h after the last training session. Animals’ performances were monitored using Any-Maze™ Video Tracking System (Stoelting Co., Wood Dale, IL), which provided data for the acquisition parameters (latency to find the platform and distance swam and) and the probe trial parameters (number of entries in the target platform zone of the platform and time in quadrants).

### Western blot analyses

RIPA extracts from mouse brain homogenates were used for western Blot analyses as previously described^14,15^. Briefly, samples were electrophoresed on 10% Bis-Tris gels or 3–8% Tris-acetate gel (Bio-Rad, Richmond, CA), transferred onto nitrocellulose membranes (Bio-Rad) and then incubated overnight at 4 °C with the appropriate primary antibodies; anti-5LO [dilution: 1:200] (Santa Cruz, Dallas, TX), anti-HT7 [1:200] (Thermo, Waltham, MA), anti-AT8 [1:100] (Thermo), anti-AT270 [1:200] (Thermo), anti-PHF13 [1:100 (Thermo)], anti-SYP [1:300] (Santa Cruz), anti-PSD95 [1:200] (Thermo), anti-GSK3α/β [1:100] (Cell Signaling, Danvers, MA), anti-pGSK3α/β [1:100] (Cell Signaling), anti-SAPK/JNK [1:100] (Cell Signaling), anti-pSAPKJNK [1:100] (Cell Signaling), anti-cdk5 1[:200] (Santa Cruz), anti-p35/p25 [1:100] (Santa Cruz), anti-PP2A [1:200] (Santa Cruz), anti-GFAP (Santa Cruz), anti-Iba1[1:100] (Thermo) and anti-Beta actin [1:500] (Santa Cruz). After three washings with T-TBS (pH 7.4), membranes were incubated with IRDye 800CW-labeled secondary antibodies (LI-COR Bioscience, Lincoln, NE) at room temperature for 1 h. Signals were developed with Odyssey Infrared Imaging Systems (LI-COR Bioscience, Lincoln, NE). β-Actin was always used as an internal loading control.

### Sarkosyl insolubility assay

The assay for insoluble tau was performed as previously described^12^. Briefly, ultracentrifugation and sarkosyl extraction (30 min in 1% sarkosyl) were used to obtain soluble and insoluble fractions of tau from brain homogenates. Insoluble fractions were washed one time with 1% sarkosyl, and then immunoblotted with the HT7 antibody.

### Immunohistochemistry

Immunostaining was performed as previously described^14,15^. Briefly, serial coronal sections were mounted on 3-aminopropyl triethoxysilane (APES)-coated slides. Every eighth section from the habenular to the posterior commissure (8–10 sections per animal) was examined with unbiased stereological principles. The sections used for testing HT7, AT8, AT270, PHF13, synaptophysin, PSD95, glial fibrillary acidic protein (GFAP) and Iba1 were deparaffinized, hydrated, rinsed with phosphate-buffered saline and pretreated with citric acid (10 mm) for 5 min for antigen retrieval, and then with 3% H_2_O_2_ in methanol for 30 min to eliminate endogenous peroxidase activity and with blocking solution (2% normal serum in Tris buffer, pH 7.6). The sections were incubated with appropriate primary antibody overnight at 4 °C, then with secondary antibody at room temperature and developed using the avidin–biotin complex method (Vector Laboratories, Burlingame, CA, USA) with 3,3-diaminobenzidine as chromogen.

### LTB_4_ assay

RIPA extracts from mouse brain homogenates from P301S and their age-matched WT counterparts were assayed for LTB_4_ levels by using a specific LTB_4_ ELISA kit (Enzo Life Sciences, Farmingdale, NY), and following the instructions of the manufacturer.

### Quantitative real-time RT-PCR

RNA from mice brain tissues was extracted and purified using the RNeasy mini-kit (Qiagen, Germantown, MD), and used as previously described^12–15^. Briefly, 1 μg of total RNA was used to synthesize cDNA in a 20 μl reaction using the RT^2^ First Strand Kit for reverse transcriptase-PCR (Super Array Bioscience). Mouse 5LO gene was amplified by using the corresponding primers obtained from Super Array Bioscience. β-Actin was used as an internal control gene to normalize for the amount of RNA. Quantitative real-time RT-PCR was performed by using Eppendorf^®^ ep realplex thermal cyclers (Eppendorf, Hauppauge, NY). Two microliters of cDNA was added to 25 μl of SYBR Green PCR Master Mix (Applied Biosystems, Foster City, CA). Each sample was run in duplicate, and analysis of relative gene expression was done by using the 2^−ΔΔCt^ method^16^. Briefly, the relative change in gene expression was calculated by subtracting the threshold cycle (ΔCt) of the target genes (5LO) from the internal control gene (β-actin). Based on the fact that the amount of cDNA doubles in each PCR cycle (assuming a PCR efficiency of 100%), the final fold-change in gene expression was calculated by using the following formula: relative change = 2^−ΔΔCt^.

### Cell line and treatment

Neuro-2A neuroblastoma (N2A) cells stably expressing human tau (N2A-tau) were cultured in Dulbecco’s modified Eagle's medium supplemented with 10% fetal bovine serum, 100 U/ml streptomycin (Mediatech, Herdon, VA) and 100 mg/ml hygromycin (Invitrogen, Carlsbad, CA) at 37 °C in the presence of 5% CO_2_. The cells were cultured to 70% confluence in six-well plates and then transfected with 200 pmol control siRNA (Santa Cruz, Dallas, TX) or 5LO siRNA (Santa Cruz) by using Lipofectamine reagent (Invitrogen) according to the manufacturer’s instructions as previously described^17^. After 72 h treatment supernatants were collected and cell harvested in lytic buffer for biochemistry analyses.

### Statistical analysis

All the data are expressed as mean ± SEM. The one-way analysis of variance test, Bonferroni multiple-comparison test and the two-tailed Student’s *t*-test were performed using Prism 5.0 (Graph Pad Software, La Jolla, CA, USA) to determine the statistical significance, with significance set at *p* < 0.05.

## Results

### Age- and region-dependent upregulation of the 5LO pathway in P301S mouse brains

**Fig. 1:**
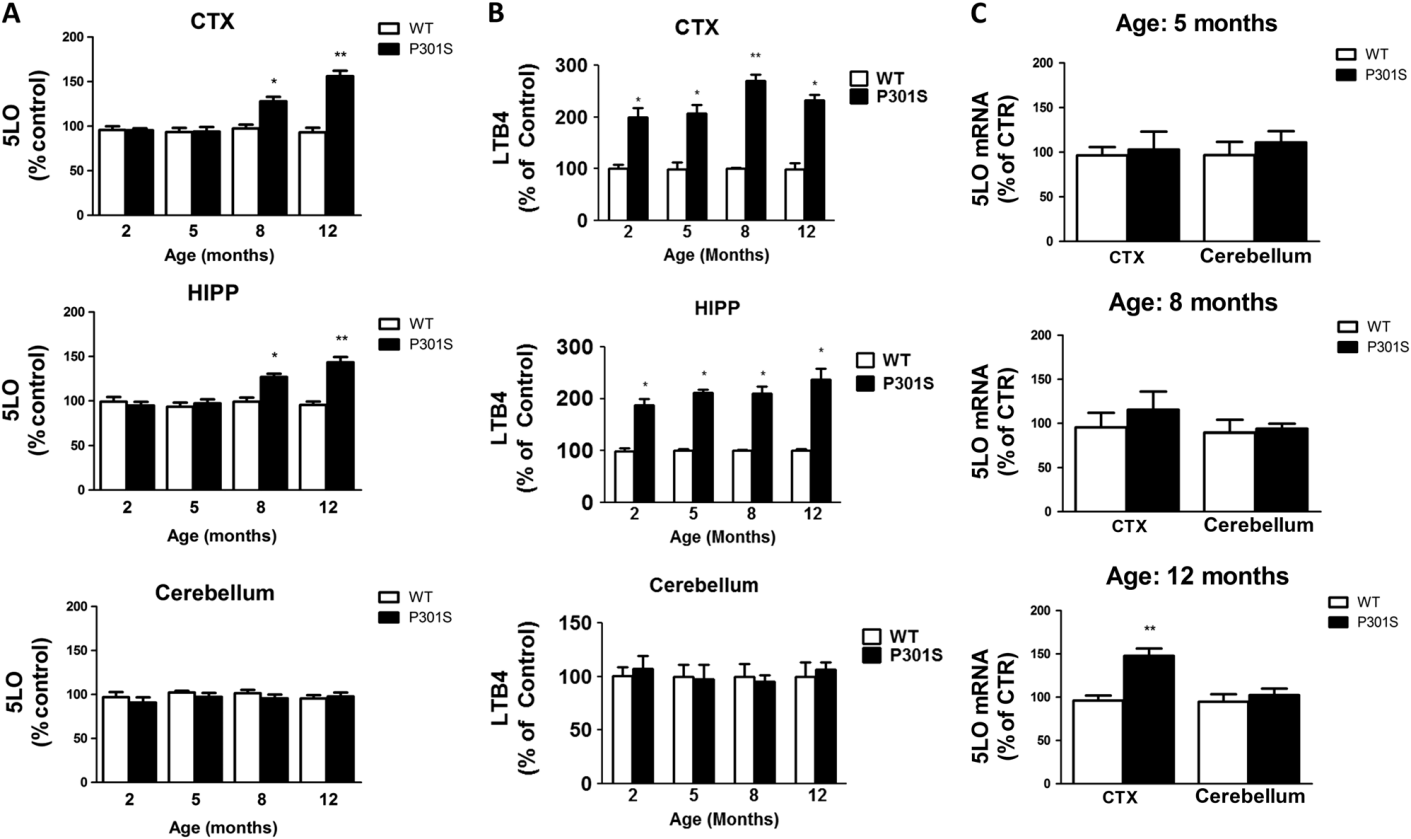
The 5LO pathway is upregulated in the brains of P301S mice in a region-specific and age-dependent manner. **a** Densitometric analyses of the western blots for 5LO protein levels in brain cortex (CTX), hippocampus (HIPP) and cerebellum homogenates from wild type (WT) and P301S mice at 2, 5, 8 and 12 months of age (*p < 0.05, **p < 0.01). **b** Levels of LTB4 measured by a sensitive and specific ELISA assay in cortex (CTX), hippocampus (HIPP) and cerebellum from WT and P301S mice at 2, 5, 8 and 12 months of age (*p < 0.01, **p < 0.001). **c** Quantitative real-time reverse transcription–polymerase chain reaction (qRT-PCR) analysis of 5LO mRNA in brain cortex (CTX) and cerebellum of WT or P301S mice at 5, 8 and 12 months of age (**p < 0.01). Results are mean ± SEM. (*n* = 6 per group)

To assess the expression levels of the 5LO pathway in the P301S mice, we first measured levels of its protein in different brain regions of these mice and matched WT controls when they were 2, 5, 8 and 12 months of age (*n* = 6 per group, per age, equal number males and females) As shown in Fig. 1a, steady-state levels of 5LO protein were significantly increased in both brain cortex and hippocampus of P301S mice at 8 and 12 months of age compared to WT controls. By contrast, no significant differences were observed between WT and P301S mice at any of the considered time points when cerebellum was assayed (Fig. 1a). To evaluate 5LO activity in the same samples, we assessed the levels of LTB4, the major metabolite produced by the activation of 5LO. In comparison to controls, P301S mice showed significantly elevated levels of LTB4 across all age time points (2, 5, 8 and 12 months) in the cortex and hippocampus, while no changes were seen in the cerebellum at any age considered (Fig. 1b). Compared with the control group, 5LO mRNA levels were significantly increased in the cortex of P301S mice at 12 months age time point, but no differences were observed at the other time points considered (Fig. 1c **)**. Levels of 5LO mRNA were no different between the two groups of mice when cerebellum was assayed (Fig. 1c).

### Genetic deletion of 5LO ameliorates cognitive deficits in P301S mice

**Fig. 2:**
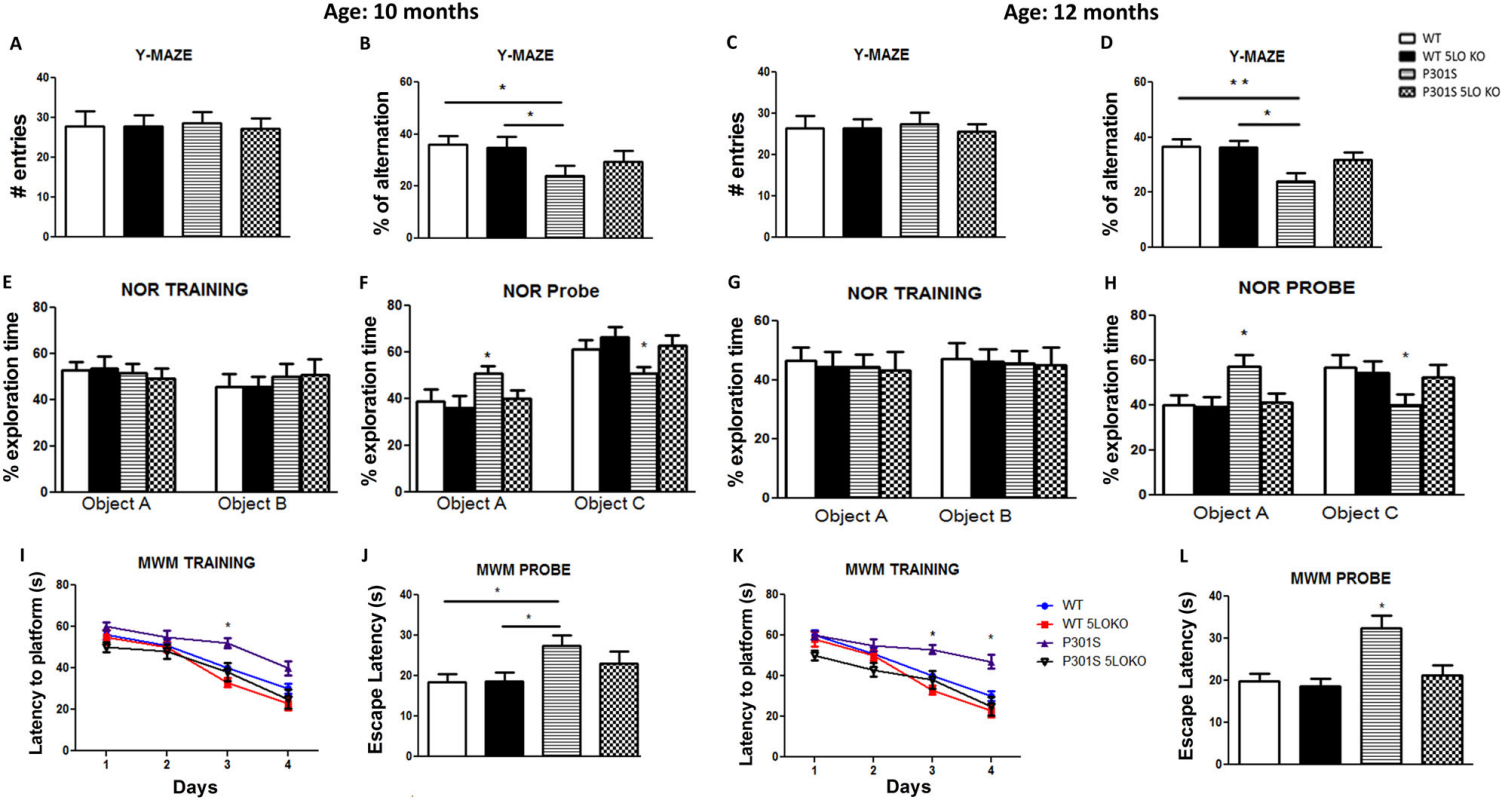
Genetic deletion of 5LO ameliorates cognitive deficits in P301S mice. **a, c** Total number of arm entries for WT, WT/5LO KO, P301S and P301S/5LO KO mice at 10 and 12 months of age during the Y-maze test. **b, d** Percentage of alternations for each of the above group of mice (*p < 0.05, **p < 0.01). **e, g** Percent exploration time during acquisition memory phase (training) of the novel object recognition test (NOR) for the four groups of mice. **f, h** Percent exploration for the novel object during retention memory phase (testing phase) for each of the above group of mice at 10 and 12 months of age (*p < 0.05). **i, k** Training phase of Morris water maze (MWM) as measured by latency to reach the platform zone at 10 and 12 months of age for each group (WT, WT/5LO KO, P301S, P301S/5LO KO), over four consecutive days (*p < 0.05). **j, l** In the probe trial we measured the latency to initial platform crossing for the four groups at 10 and 12 months of age (*p < 0.05). Values are expressed as mean ± SEM (*n* = 9–10 in each 10-month group, *n* = 7–8 in each 12-month group)

Since our findings indicate an early upregulation of the 5LO pathway in this mouse model of tauopathy, next we tested whether its genetic absence would affect the development of its phenotype. To this end, we generated P301S mice genetically deficient for the 5LO gene, P301S/5LO KO mice, and compared them with the regular P301S (5LO+/+), WT controls and WT lacking the 5LO (WT 5LO KO) in the following behavioral paradigms: Y-maze, novel object recognition, and Morris water maze at two different age time points: 10 and 12 months. As shown in Fig. 2a and c no differences were observed in regard to total number of entries into each arm at either time points (10 and 12 months) among the four groups of mice. Compared to WT and WT 5LO KO, P301S mice show a decreased percentage of arm alternations in this paradigm at both 10 and 12 months. However, despite the fact that P301S/5LO KO mice showed improvement in the percentage alternation at both ages, the difference did not reach statistical significance (Fig. 2b, d). As a second measure of explorative and working memory, mice were tested in the novel object recognition (NOR) paradigm. During the training phase, no differences among the four groups of mice were observed at both ages, indicating that mice did not show preference for one object over another, or one side of the cage (Fig. 2e, g). However, during the probe trial, compared with WT controls with and without the 5LO, the P301S mice did not show preference for the novel object compared to the original object (Fig. 2f, h **)**. This effect was rescued in P301S/5LO KO mice, which showed preference for the novel object with exploration percentages that were comparable to WT, indicating an amelioration of working memory in these mice for both age time points (Fig. 2f, h). Mice were also tested for spatial learning and memory as measured by the Morris water maze paradigm. All mice swam with similar proficiency and were able to visually find the platform (data not shown). During the training phase, P301S performed worse than WT mice with and without the 5LO on day 3 (10- and 12-month time points) and day 4 (12-month time point) (Fig. 2i, k). However, P301S/5LO KO learned in a manner indistinguishable from both WT groups at both time points (Fig. 2i, k). In the probe trial, compared with both WT and WT/5LO KO, P301S mice displayed increased latency to cross the platform zone at both 10 and 12 months, which was absent in P301S 5LO KO mice (Fig. 2j, l). Finally, the time spent in the platform zone remained unchanged among the different groups both at 10 and 12 months of age (not shown).

### 5LO genetic absence modulates tau phosphorylation and pathology in P301S mice

**Fig. 3:**
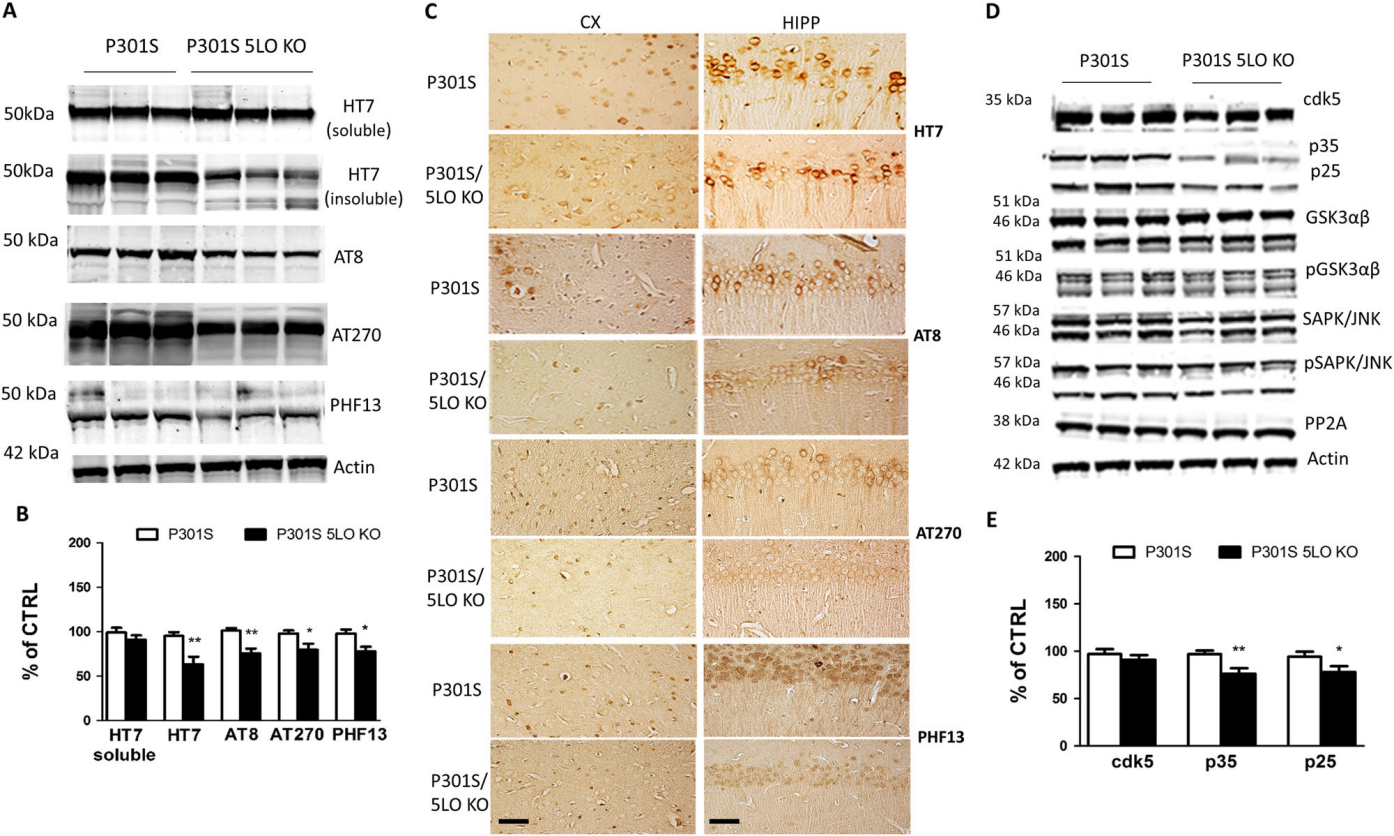
Genetic absence of 5LO modulates tau phosphorylation in P301S mice. **a** Representative western blot analyses for soluble tau and insoluble tau (HT7) and phosphorylated tau at residues Ser202/Thr205 (AT8), Thr181 (AT270) and Ser396 (PHF13) in brain cortex homogenates from P301S and P301S/5LO KO mice at 12 months of age. **b** Densitometric analyses of the immunoreactivities to the antibodies shown in panel **a** (*p < 0.05). Results are mean ± SEM (*n* = 6 per group). **c** Representative images of immunohistochemical staining of the cortex (CX) and hippocampus (HIPP) (CA1) region of P301S and P301S/5LO KO mice for HT7, AT8, AT270 and PHF13 antibodies (scale bar = 50 μm). **d** Representative western blot analyses for cyclin-dependent kinase (cdk)5, p35, p25, glycogen synthase kinase (GSK3α, GSK3α, pGSK3α, pGSK3β), stress-activated protein kinase/jun amino terminal kinase (SAPK/JNK1, SAPK/JNK2, p-SAPK/JNK1, p-SAPK/JNK2) and phosphatase protein-2 (PP2)A protein levels in brain cortex homogenates from P301S and P301S/5LO KO mice. **e** Densitometric analyses of the immunoreactivities to the antibodies from panel **d** (*p < 0.05, **p < 0.01; *n* = 5 per group). Results are mean ± SEM

Next, we assessed the effect of 5LO genetic deletion on tau phosphorylation and pathology. To this end, we measured protein levels of total tau and its phosphorylated isoforms at different epitopes in brain cortex homogenates from P301S and P301S/5LO KO mice. As shown in Fig. 3a, levels of total soluble tau remained unchanged when the two groups of mice were compared. However, P301S/5LO KO mice showed a reduction in phosphorylated tau at Ser202/Thr205, Thr181 and Ser396, as recognized by the antibodies AT8, AT270 and PHF13, respectively (Fig. 3a, b). Confirming the western blot data, histochemical staining showed a reduction in phosphorylated tau epitopes immunoreactivity in the brains of P301S/5LO KO mice compared to controls, while no changes were seen in total soluble tau **(**Fig. 3c **)**. Additionally, we examined levels of insoluble tau in P301S mice depleted of 5LO and found a significant decrease in its levels when compared with P301S mice (Fig. 3a, b).

To elucidate the potential mechanism that may be driving the changes in tau phosphorylation in our mouse model, we measured the levels of some key kinases that are responsible for phosphorylating tau. When comparing P301S and P301S/5LO KO, while we observed no changes in levels of cdk5 kinase, a significant reduction in both of its co-activators p35/p25 was detected (Fig. 3d, e). By contrast, no differences between the two groups were found for the total or phosphorylated glycogen synthase kinase 3-α (GSK3-α) and GSK3-β, total and phosphorylated stress-activated protein kinase (SAPK/JNK), and protein phosphatase 2A (PP2A) (Fig. 3d).

### Synaptic pathology in P301S mice is rescued by genetic absence of 5LO

**Fig. 4:**
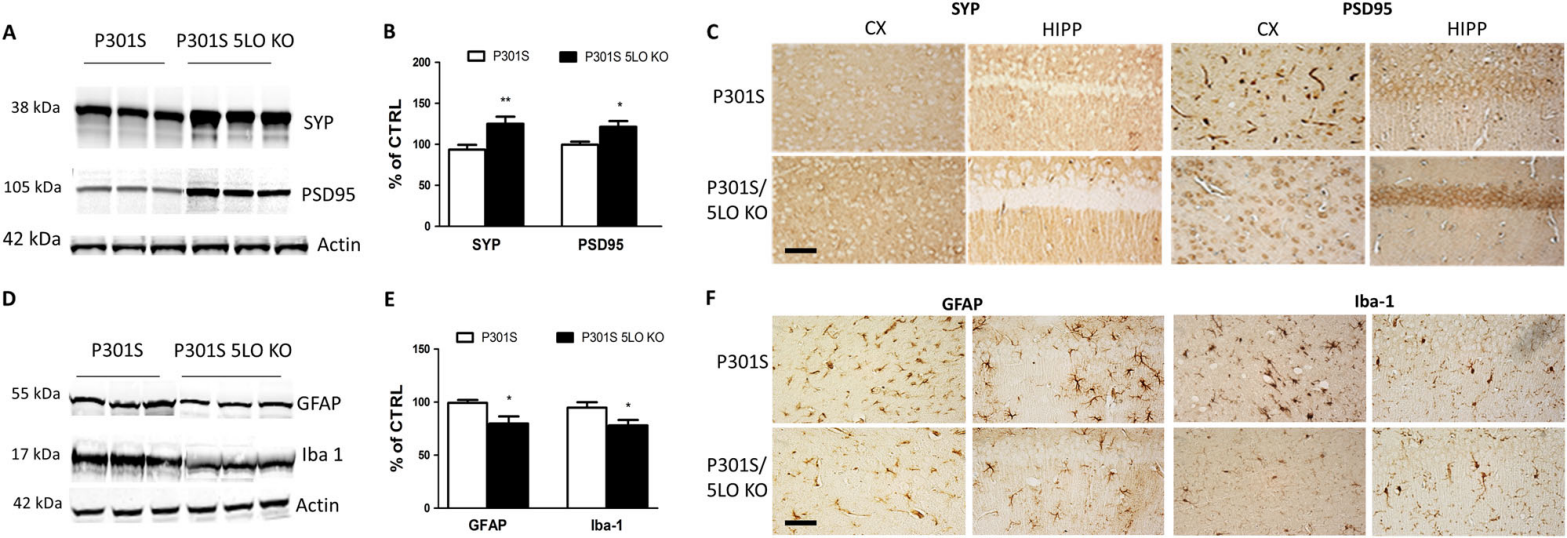
Genetic absence of 5LO ameliorates synaptic pathology and neuroinflammation in P301S mice. **a** Representative western blot analyses for synaptophysin (SYP) and postsynaptic density protein-95 (PSD95) in brain cortex homogenates from P301S and P301S/5LO KO mice at 12 months. **b** Densitometric analyses of the immunoreactivities shown in the previous panel (*p < 0.05, **p < 0.01) (*n* = 5 per group). **c** Representative images of immunohistochemical staining in the cortex (CX) and hippocampus (HIPP) (CA1 region) for SYP and PSD95 in P301S and P301S/5LO KO mice at 12 months of age. **d** Representative western blot analyses for glial fibrillary acidic protein (GFAP) and ionized calcium-binding adapter molecule 1 (Iba1) in brain cortex homogenates from P301S and P301S/5LO KO mice at 12 months. **e** Densitometric analyses of the immunoreactivities shown in the previous panel (*p < 0.05) (*n* = 4 per group). **f** Representative images of immunohistochemical staining in the cortex (CX) and hippocampus (HIPP) (CA1 region) for GFAP and Iba1 in P301S and P301S/5LO KO mice at 12 months of age (scale bar = 50μm)

Modifications in synaptic integrity often follow alterations in tau phosphorylation and tangles formation and usually associate with cognitive changes. Thus, we investigated whether biomarkers of synaptic integrity were affected by 5LO absence in P301S mice. Compared to P301S mice, the P301S/5LO KO mice display enhanced steady-state levels of two major synaptic proteins: synaptophysin and postsynaptic density protein-95 (PSD95) **(**Fig. 4a, b **)**. These results were also supported by the findings of the immunohistochemical analyses on brains sections from the two groups of mice showing an increase in the reactivity for both synaptophysin and PSD95 proteins in brain sections from P301S/5LO KO versus P301S mice (Fig. 4c).

### Neuroinflammation in P301S mice is modulated by 5LO

Compared with controls, P301/5LO KO mice had a significant decrease in the steady-state levels of the GFAP, a marker of astrocytes activation and ionized calcium-binding adapter molecule 1 (Iba1), a marker of microglia activation; both indicators of neuroinflammatory reactions (Fig. 4d, e). Immunohistochemical analyses confirmed our western blot findings, showing a decrease in reactivity for GFAP and Iba1 protein (Fig. 4f).

### Downregulation of 5LO reduces tau phosphorylation

**Fig. 5:**
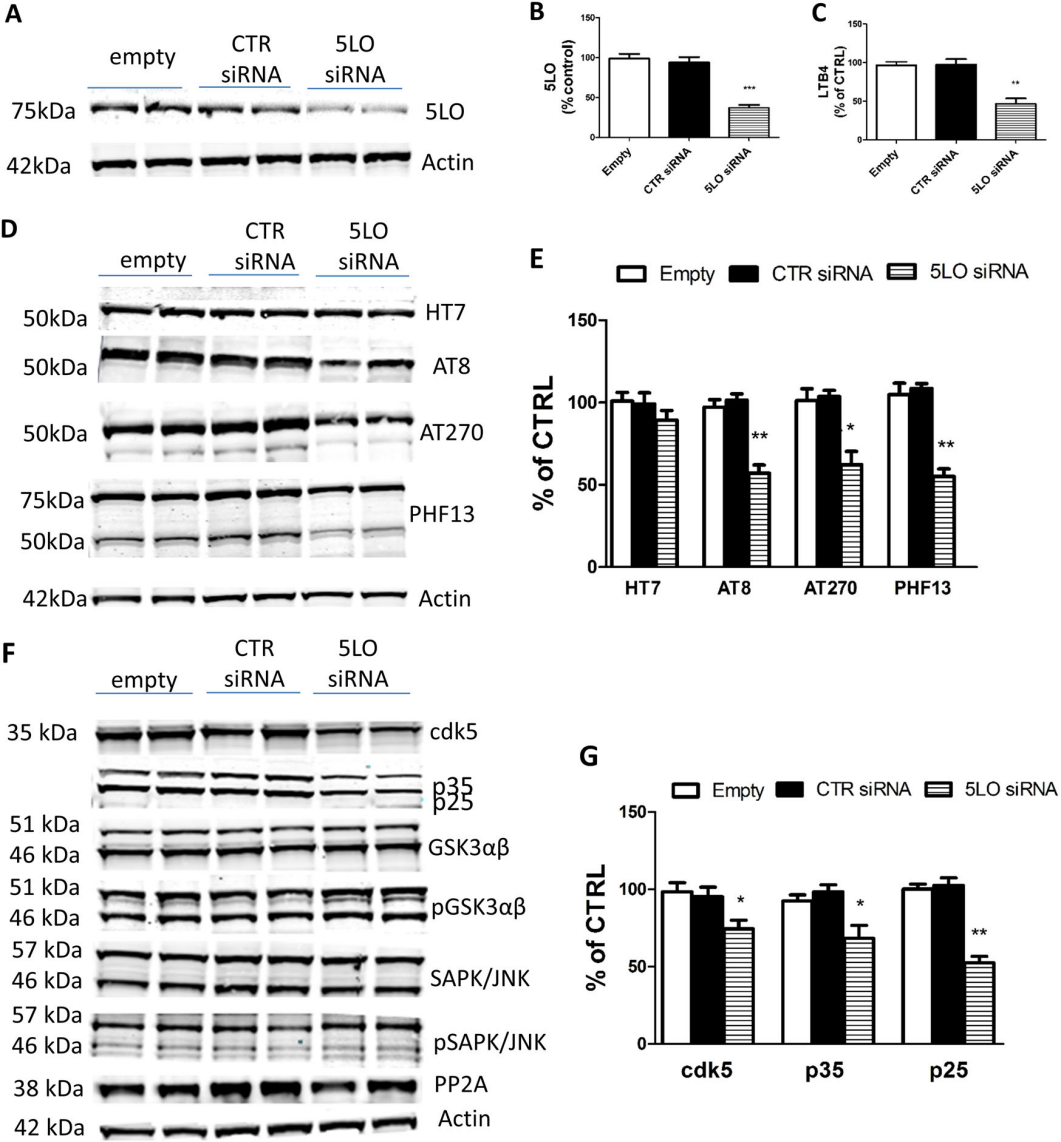
Downregulation of 5LO decreases tau phosphorylation. N2A neuronal cells stably expressing human tau protein were transiently transfected with empty vector, control siRNA or 5LO siRNA for 72 h, and supernatants and cells lysates harvested for biochemistry. **a** Representative western blot analysis for 5LO protein in cells lysates transfected with mouse 5LO siRNA (200 pmol), empty vector or control siRNA (CTR). **b** Densitometric analyses of the immunoreactivity to the antibody shown in previous panel (***p < 0.001). **c** Levels of LTB4 in conditioned media from the same cells described in the previous panel (**p < 0.01). **d** Representative western blot analysis for total tau (HT7) and phosphorylated tau at residues Ser202/Thr205 (AT8), Thr181 (AT270) and Ser396 (PHF13) in lysates from the same cells. **e** Densitometric analyses of the immunoreactivities to the antibodies shown in panel **d** (*p < 0.05, **p < 0.01). **f** Representative western blot analyses cdk5, p35 and p25, glycogen synthase kinase (GSK3α, GSK3α, pGSK3α, pGSK3β), stress activated protein kinase/jun amino terminal kinase (SAPK/JNK1, SAPK/JNK2, p-SAPK/JNK1, p-SAPK/JNK2) and phosphatase protein-2 (PP2)A protein levels in lysates from 5LO siRNA or control transfected neuronal cells. **g** Densitometric analyses of the immunoreactivities to the antibodies for cdk5, p35 and p25 shown in panel **e** (*p < 0.05, **p < 0.01) Results are mean ± SEM (*n* = 3 per conditions, in duplicate)

N2A cells stably expressing human tau were transiently transfected with 5LO siRNA, empty vector or control siRNA for 72 h. At this time point, compared with control cells, cells receiving 5LO siRNA had a significant decrease in the steady-state levels of 5LO protein, which was accompanied by a similar reduction in its enzymatic activity, as shown by the lower levels of LTB4 in the supernatant (Fig. 5a–c). No changes were observed for the amount of total tau protein between the two groups. However, we observed that cells with 5LO downregulation had a statistically significant decrease in the phosphorylated tau isoforms at epitopes Ser202/Thr205, Thr181 and Ser396, as recognized by the antibodies AT8, AT270 and PHF13, respectively (Fig. 5d, e). Under this experimental condition, we observed that steady-state levels of cdk5 as well as the levels of its two main co-activators, p35 and p25, were significantly reduced in the same cells (Fig. 5f, g).

## Discussion

The current study provides experimental evidence for a functional role of the 5LO pathway in the development of the tau pathological phenotype, which includes increased tau phosphorylation, neuroinflammation, synaptic pathology and cognitive impairments in a relevant mouse model of human tauopathy, the P301S mice.

The term ‘tauopathy’ typically refers to a heterogeneous group of neurodegenerative diseases and clinical syndromes having a specific and prevalent pathological brain signature: progressive accumulation of highly phosphorylated and insoluble tau protein deposits, typically referred to as neurofibrillary tangles^18,19^. Today, while we know that a small percentage of these diseases are secondary to autosomic mutations of genes involved in tau metabolism, for the majority of them, known as sporadic tauopathies, a combination of environmental factors and genetic risk factors have been invoked as active contributors to their pathogenesis^20^.

In recent years, the 5LO enzymatic pathway has emerged as a novel candidate gene for neurodegeneration, since, by controlling inflammatory reactions, it acts as an endogenous modulator of brain amyloidosis in transgenic mouse models overexpressing human APP variant^21^, as well as in the development of the AD-like phenotype of the triple-transgenic mouse model, which develops both Aβ plaques and tau tangles^22^. Moreover, we have shown that steady-state levels of 5LO are increased in brain frontal cortices from subjects with a post-mortem diagnosis of PSP and that pharmacologic blockade of the 5LO activation ameliorates tau pathology and behavioral deficits in a mouse model expressing human tau, the h-tau mice^23^.

However, despite the specificity and selectivity of the drug implemented in that study to irreversibly block 5LO enzymatic activation, as for any other pharmacological approach, ours is not immune from potential “off target” effects. Therefore, in order to gain further support for the involvement of this enzymatic pathway in the development of the tau pathological phenotype, in the current paper we used a genetic approach targeting 5LO and a different mouse model of tauopathy, the P301S mice.

First, we wanted to investigate the expression levels of the 5LO pathway and how its potential changes were related to the age-dependent development of the tau phenotype by assessing 5LO protein, enzymatic activity and mRNA levels in different brain regions and at different age time points in these mice. Compared to WT counterparts, P301S mice showed a significant age-dependent increase in the steady-state levels of 5LO protein both in the cortex and hippocampus, two brain regions known to be vulnerable to neurodegenerative insults and areas of tau pathology accumulation^24,25^. By contrast, we did not observe any differences in 5LO protein levels across age groups when we assayed the cerebellum, an area typically void of any tau pathology^24,25^. Taken together these results suggest not only an age-dependency but also a region-specificity for the expression levels of 5LO protein in this mouse model. Interestingly, when we checked for the activity of 5LO by measuring levels of LTB4, the main metabolic product of 5LO enzyme activation, we discovered that, compared with WT controls, LTB4 levels were significantly elevated as early as at 2 months of age both in the cortical as well as hippocampal brain regions. By contrast, no significant differences were found in the cerebellum when the two groups of mice were compared. These findings support the novel hypothesis that, in the P301S transgenic mouse model of human tauopathy, the activation of the 5LO pathway is an early event during the development of their phenotype, since it is evident at such a very young age, 2-month-old, when no tau pathology is detectable. On the basis of this initial observation, next we asked whether 5LO plays a functional role in modulating tau phosphorylation, neuroinflammation, synaptic pathology and behavioral deficits in these mice by using a genetic approach.

Herein, we demonstrate that compared with P301S mice having 5LO, the genetic absence of this protein in the same mice results in a significant reduction of tau phosphorylation and neuropathology. This reduction is associated with an improvement of behavioral responses in the Y-maze and NOR paradigms, measurements of working and exploratory memory, as well as the MWM, a measure of spatial and learning memory. Thus, when compared with WT, the P301S mice had a lower number of alternations in the Y-maze, explored the novel object less in the NOR test and manifested higher escape latency to the platform in the MWM. By contrast, the genetic absence of 5LO in P301S mice was sufficient to rescue this behavioral phenotype. It is important to note that all of these results were not secondary to an effect of the genetic deficiency of 5LO per se on the motor ability of the mice investigated, since no differences among the four groups were observed in the number of entries for the Y-maze, the training results for the NOR, and the swimming speed for the MWM.

Since learning and memory task responses are known to be directly modulated by synaptic function and integrity, next we assessed two well-established synaptic markers, synaptophysin and PSD95^26,27^. Compared to P301S, a significant amelioration of synaptic integrity was found in our P301S/5LO KO model as shown by the higher levels of both proteins.

Under our experimental condition, we observed that 5LO absence had no effect on the levels of total tau, which were indistinguishable between P301S with and without the gene. This fact rules out that the observed effects on tau phosphorylation were secondary to some unpredictable consequence of the 5LO genetic deletion on the tau transgene. It is of great interest that the effect on tau phosphorylation was also specific since we observed a significant reduction on particular tau epitopes such as Ser202/Thr205, Thr181 and Ser396, which have been linked to the development of neurofibrillary tangles in both AD and related tauopathies^28,29^. To dissect a potential mechanism for the changes we observed in tau phosphorylation, we assayed some of the key kinases responsible for phosphorylating tau as well as the phosphatase PP2A, and found that the effect on tau phosphorylation was associated with a reduction only for the ckd5 kinase pathway.

Having demonstrated a significant reduction in tau phosphorylation and tau solubility, next we assessed whether neuroinflammatory markers were also affected by the 5LO genetic absence in our model. To this end we focus on GFAP and Iba1 proteins, markers of astrocyte and microglia activation, respectively, which when elevated are considered the biochemical signature of dysregulated neuroinflammatory reactions^30,31^. Considering that this protein is a pro-inflammatory enzyme which produces potent bioactive lipid mediators, it was not surprising to find that both markers were significantly reduced in the brains of P301S/5LO KO mice when compared with regular P301S.

Finally, our in vivo findings were confirmed by using an in vitro approach recapitulating our mouse model and characterized by neuronal cells stably expressing human tau protein in which 5LO levels were downregulated. Similar to the results we obtained in vivo with P301S/5LO KO mice, compared with control cells, 5LO silencing, which was confirmed by a significant reduction in 5LO protein levels and activity, manifested a significant decrease in the phosphorylation of tau proteins at the same specific epitopes as our in vivo experiments, but no changes in the amount of total tau. Additionally, these changes were associated with lower levels of the cdk5 kinase pathway.

Since in our model the genetic absence of 5LO is not restricted to the central nervous system but generalized, it is possible that the peripheral absence of this enzyme could also in part contribute to the effects. However, considering that 5LO is upregulated in the brains of our mouse model and that its metabolites act only locally, we believe that the absence of peripheral 5LO was not involved in the observed biological effects in the P301S mice.

In conclusion, taken together our findings demonstrate a direct role of the 5LO pathway in modulating tau phosphorylation and neuropathology in the P301S mouse model. They support the novel concept that the upregulation of 5LO in the central nervous system of this transgenic mouse model of human tauopathy not only is an early event in the pathogenesis of the disease, but an important player with an active role in the development of the entire pathological phenotype. Our data provide further experimental support and strong preclinical evidence that this pathway should be considered as a viable pharmacological target for treating and or halting human tauopathies.

## References

[1] Giannopoulos, F. G. & Pratico, D. In: *Diet and Nutrition in Dementia and Cognitive Decline* (eds Martin, C. R. & Reddy V.) 13–21 (Elseveier Publisher, London, UK, 2015).

[2] Spillantini MG, Goedert M (2013). Tau pathology and neurodegeneration. Lancet Neurol..

[3] Wang Y, Mandelkow E (2016). Tau in physiology and pathology. Nat. Rev. Neurosci..

[4] Yoshiyama Y (2007). Synapse loss and microglial activation precede tangles in a P301S tauopathy mouse model. Neuron.

[5] Mandrekar-Colucci S, Landreth GE (2010). Microglia and inflammation in Alzheimer’s disease. CNS Neurol. Disord. Drug Targets.

[6] Laurent C (2017). Hippocampal T cell infiltration promotes neuroinflammation and cognitive decline in a mouse model of tauopathy. Brain.

[7] Chinnici CM, Yao Y, Praticò D (2007). The 5-lipoxygenase enzymatic pathway in the mouse brain: young versus old. Neurobiol. Aging.

[8] Firuzi O, Zhuo J, Chinnici CM, Wisniewski T, Praticò D (2008). 5-Lipoxygenase gene disruption reduces amyloid-beta pathology in a mouse model of Alzheimer’s disease. FASEB J..

[9] Chu J, Giannopoulos PF, Ceballos-Diaz C, Golde TE, Pratico D (2012). Adeno-associated virus-mediated brain delivery of 5-lipoxygenase modulates the AD-like phenotype of APP mice. Mol. Neurodegener..

[10] Chu J, Praticò D (2011). Pharmacologic blockade of 5-lipoxygenase improves the amyloidotic phenotype of an Alzheimer’s disease transgenic mouse model involvement of γ-secretase. Am. J. Pathol..

[11] Giannopoulos PF (2014). Gene knockout of 5-lipoxygenase rescues synaptic dysfunction and improves memory in the triple-transgenic model of Alzheimer’s disease. Mol. Psychiatry.

[12] Li JG, Chu J, Barrero C, Merali S, Praticò D (2014). Homocysteine exacerbates β-amyloid pathology, tau pathology, and cognitive deficit in a mouse model of Alzheimer disease with plaques and tangles. Ann. Neurol..

[13] Di Meco A, Lauretti E, Vagnozzi AN, Praticò D (2014). Zileuton restores memory impairments and reverses amyloid and tau pathology in aged Alzheimer’s disease mice. Neurobiol. Aging.

[14] Di Meco A (2017). 12/15-Lipoxygenase inhibition reverses cognitive impairment, brain amyloidosis, and tau pathology by stimulating autophagy in aged triple transgenic mice. Biol. Psychiatry.

[15] Lauretti E, Li JG, Di Meco A, Praticò D (2017). Glucose deficit triggers tau pathology and synaptic dysfunction in a tauopathy mouse model. Transl. Psychiatry.

[16] Livak KJ, Schmittgen TD (2001). Analysis of relative gene expression data using real-time quantitative PCR and the 2(-Delta Delta C(T)) method. Methods.

[17] Chu J, Giannopoulos PF, Ceballos-Diaz C, Golde TE, Praticò D (2012). 5-Lipoxygenase gene transfer worsens memory, amyloid, and tau brain pathologies in a mouse model of Alzheimer disease. Ann. Neurol..

[18] Arendt T, Stieler JT, Holzer M (2016). Tau and tauopathies. Brain. Res. Bull..

[19] Lamb R, Rohrer JD, Lees AJ, Morris HR (2016). Progressive supranuclear palsy and corticobasal degeneration: pathophysiology and treatment options. Curr.Treat. Options Neurol..

[20] Williams DR (2006). Tauopathies: classification and clinical update on neurodegenerative diseases associated with microtubule-associated protein tau. Intern. Med. J..

[21] Chu J, Praticò D (2011). 5-Lipoxygenase as an endogenous modulator of amyloid-β formation in vivo. Ann. Neurol..

[22] Joshi YB (2014). Absence of ALOX5 gene prevents stress-induced memory deficits, synaptic dysfunction and tauopathy in a mouse model of Alzheimer’s disease. Hum. Mol. Genet..

[23] Giannopoulos PF (2015). Pharmacologic inhibition of 5-lipoxygenase improves memory, rescues synaptic dysfunction, and ameliorates tau pathology in a transgenic model of tauopathy. Biol. Psychiatry.

[24] Yang S, Kuan WL, Spillantini MG (2016). Progressive tauopathy in P301S tau transgenic mice is associated with a functional deficit of the olfactory system. Eur. J. Neurosci..

[25] Allen B (2002). Abundant tau filaments and nonapoptotic neurodegeneration in transgenic mice expressing human P301S tau protein. J. Neurosci..

[26] Hoover BR (2010). Tau mislocalization to dendritic spines mediates synaptic dysfunction independently of neurodegeneration. Neuron.

[27] Sydow A (2011). Tau-induced defects in synaptic plasticity, learning, and memory are reversible in transgenic mice after switching off the toxic Tau mutant. J. Neurosci..

[28] Buée L, Bussière T, Buée-Scherrer V, Delacourte A, Hof PR (2000). Tau protein isoforms, phosphorylation and role in neurodegenerative disorders. Brain. Res. Brain. Res. Rev..

[29] Wang JZ, Xia YY, Grundke-Iqbal I, Iqbal K (2013). Abnormal hyperphosphorylation of tau: sites, regulation, and molecular mechanism of neurofibrillary degeneration. J. Alzheimers Dis..

[30] Bellucci A (2004). Induction of inflammatory mediators and microglial activation in mice transgenic for mutant human P301S tau protein. Am. J. Pathol..

[31] McGeer EG, McGeer PL (2010). Neuroinflammation in Alzheimer’s disease and mild cognitive impairment: a field in its infancy. J. Alzheimers Dis..

